# Adult Non-Cystic Fibrosis Bronchiectasis Is Characterised by Airway Luminal Th17 Pathway Activation

**DOI:** 10.1371/journal.pone.0119325

**Published:** 2015-03-30

**Authors:** Alice C.-H. Chen, Megan L. Martin, Rohan Lourie, Geraint B. Rogers, Lucy D. Burr, Sumaira Z. Hasnain, Simon D. Bowler, Michael A. McGuckin, David J. Serisier

**Affiliations:** 1 Immunity, Infection and Inflammation Program, Mater Research—University of Qld, Translational Research Institute, Woolloongabba, Qld, Australia; 2 Department of Respiratory Medicine, Mater Adult Hospital, South Brisbane, Qld, Australia; 3 Department of Anatomical Pathology, Mater Health Services, South Brisbane, Qld, Australia; 4 Infection and Immunity Theme, South Australia Health and Medical Research Institute, North Terrace, Adelaide, Australia; 5 School of Medicine, Flinders University, Bedford Park, Adelaide, Australia; 6 School of Biomedical Science, The University of Queensland, Qld, Australia; University Medical Center Utrecht, NETHERLANDS

## Abstract

**Background:**

Non-cystic fibrosis (CF) bronchiectasis is characterised by chronic airway infection and neutrophilic inflammation, which we hypothesised would be associated with Th17 pathway activation.

**Methods:**

Th17 pathway cytokines were quantified in bronchoalveolar lavage fluid (BALF), and gene expression of IL-17A, IL-1β, IL-8 and IL-23 determined from endobronchial biopsies (EBx) in 41 stable bronchiectasis subjects and 20 healthy controls. Relationships between IL-17A levels and infection status, important clinical measures and subsequent *Pseudomonas aeruginosa* infection were determined.

**Results:**

BALF levels of all Th17 cytokines (median (IQR) pg/mL) were significantly higher in bronchiectasis than control subjects, including IL-17A (1.73 (1.19, 3.23) vs. 0.27 (0.24, 0.35), 95% CI 1.05 to 2.21, p<0.0001) and IL-23 (9.48 (4.79, 15.75) vs. 0.70 (0.43, 1.79), 95% CI 4.68 to 11.21, p<0.0001). However, BALF IL-17A levels were not associated with clinical measures or airway microbiology, nor predictive of subsequent P. aeruginosa infection. Furthermore, gene expression of IL-17A in bronchiectasis EBx did not differ from control. In contrast, gene expression (relative to medians of controls) in bronchiectasis EBx was significantly higher than control for IL1β (4.12 (1.24, 8.05) vs 1 (0.13, 2.95), 95% CI 0.05 to 4.07, p = 0.04) and IL-8 (3.75 (1.64, 11.27) vs 1 (0.54, 3.89), 95% CI 0.32 to 4.87, p = 0.02) and BALF IL-8 and IL-1α levels showed significant relationships with clinical measures and airway microbiology. *P*. *aeruginosa* infection was associated with increased levels of IL-8 while *Haemophilus influenzae* was associated with increased IL-1α.

**Conclusions and Clinical Relevance:**

Established adult non-CF bronchiectasis is characterised by luminal Th17 pathway activation, however this pathway may be relatively less important than activation of non-antigen-specific innate neutrophilic immunity.

## Introduction

Bronchiectasis is a condition characterised by permanent bronchial dilatation which can result from a myriad of underlying aetiologies, although most cases are idiopathic [1]. The prevalence of non-CF bronchiectasis (hereafter bronchiectasis) is poorly characterised, however it is frequently encountered in clinical practice and emerging evidence suggests a greater prevalence than previously appreciated [2]. Additionally, bronchiectasis is a common respiratory co-pathology, present in at least 30% of those with smoking-related chronic obstructive pulmonary disease (COPD) [3]. Furthermore, recent data suggest that the co-existence of bronchiectasis in COPD subjects is independently associated with a more than doubling in the risk of death [4].

Airway inflammation in bronchiectasis is characterised by neutrophil-dominated inflammation associated with elevated levels of proinflammatory cytokines and chemokines such as IL-8, IL-6 and TNF-α in bronchoalveolar lavage fluid (BALF)[5]. Furthermore, significant relationships have been shown to exist between airway inflammation and microbiology assessed by both culture and molecular methods [5–7], supporting Cole’s proposed ‘vicious cycle’ hypothesis [8]. Neutrophil-predominant acute inflammation is typical of the inflammatory response associated with the antigen-specific Th17 T cell pathway, whose primary function appears to be clearance of extracellular bacterial and fungal pathogens, and hence this pathway is likely to be involved in bronchiectasis [9]. The major product of Th17 cells, IL-17A, stimulates local cytokine (eg IL-1β, TNF-α and IL-6) and chemokine (eg CXCL1, CXCL2, CXCL5 and CXCL8/IL-8) secretion by other cells, inducing neutrophil recruitment and activation [9]. Additionally, IL-17 induces the release of matrix metalloproteinases (MMPs) from various cell types, resulting in matrix destruction [9]. MMP’s have been shown to be elevated in bronchiectasis and may be important in the processes leading to bronchiectasis itself [10].

Recent studies have demonstrated elevated levels of IL-17A in BALF from children with CF [11,12]. In CF children, elevated BALF IL-17A levels at baseline were associated with subsequent acquisition of *Pseudomonas aeruginosa* infection [12], and increases in cells staining positive for IL-17 have been identified in airway mucosal biopsies from both CF and non-CF bronchiectasis children [11]. The potential involvement of this pathway in adults with established bronchiectasis remains incompletely defined, limited to a single study in which IL-17 was detected in exhaled breath condensate [13].

As part of the Bronchiectasis and Low-dose Erythromycin Study (BLESS) [14], we evaluated the Th17 pathway in a subgroup of subjects who underwent bronchoscopy at baseline. We hypothesized that subjects with non-CF bronchiectasis would demonstrate significant activation of the Th17 pathway. Furthermore, we sought to evaluate potential associations between baseline BALF IL-17A and airway infection by pathogens, particularly *P*. *aeruginosa*, and the development of new *P*. *aeruginosa* infection in the 12 months following sampling.

## Methods

### Subjects and procedures (further details in S1 Supporting Information for all sections)

This study was approved by the Mater Health Services Human Research Ethics Committee (1244A) and all subjects provided written, informed consent. All sample processing and evaluation was undertaken with complete blinding to disease state and study treatment assignment. A subgroup of 41 participants randomised in the BLESS study [14] underwent bronchoscopic sampling at baseline, prior to commencing assigned trial medication. Subjects in this subgroup had no evidence of additional (non-bronchiectasis) chronic respiratory disease. The normal control group comprised 20 healthy volunteers recruited from hospital and research institute staff. All bronchoscopies were performed by the same proceduralist (DJS). All control and bronchiectasis subjects were macrolide-naïve, lifelong non-smokers (<2 pack year history), aged 18–85 years, with no history of asthma or atopy, recent respiratory tract infection (within 4 weeks) or conditions with the potential to impact the safe performance of bronchoscopy. (Full study details including inclusion and exclusion criteria are provided in S1 Supporting Information).

Bronchoscopy was performed as an outpatient procedure under conscious sedation. After the application of lignocaine to the airways, BAL was first performed in the right middle lobe and then endobronchial biopsies collected from subsegmental carinae of the lower lobes. BAL fluid (BALF) samples were placed on ice and processed immediately following the procedure.

At screening, control and bronchiectasis subjects had demographic data collected and completed spirometry before and after inhalation of salbutamol bronchodilator. Bronchiectasis subjects provided a 24 hour sputum sample, completed symptom (Leicester Cough/ LCQ) and quality of life (QOL; St George’s Respiratory/ SGRQ) questionnaires, produced a sputum sample for microbiology and completed a 6-minute walk test (6MWT) [14]. (Further details are provided in S1 Supporting Information).

### Gene expression from endobronchial biopsies

Two endobronchial biopsies for RNA extraction were placed into a tube containing 0.5 mL of RNA Later solution (Life Technologies, Carlsbad, CA) and stored at -80°C for subsequent processing. RNA was extracted from biopsies and amplified (Ovation Amplification Kit, NuGen, San Carlos, CA) prior to analysis of gene expression of IL-1β, IL-8, IL-17A and IL-23 by TaqMan qRT-PCR (Microfluidic Cards, Life Technologies, Carlsbad, CA) as per the manufacturer’s instructions. All qRT-PCR results were corrected for a group of three house-keeping genes.

### BALF processing

The BALF sample was separated into 1 mL aliquots and immediately placed on ice. One sample was sent to the microbiology laboratory, a second sample was processed within 30 minutes for differential cell counts, and a third processed within 60 minutes to generate supernatant for cytokine analysis.

### Cytokine and chemokine analysis

The Procarta Luminex cytokine and chemokine array (Affymetrix, Santa Clara, CA) was used to determine concentrations of IL-1α, IL-1β, IL-6, IL-8, IL-17A, IL-23 and TNF- α in 50 μL concentrated (by 10kDa cut-off centrifugal concentrator) BALF supernatant with protease inhibitor. Reported results have been converted back to original concentrations per mL of BALF.

### Data analysis

Sample size calculations for the BLESS study are as described previously [14,15]. Data distribution was mostly non-parametric and Mann-Whitney U tests were used to test the significance of variance between control and disease groups, or Kruskal-Wallis test where 3 or more groups were compared (with post-hoc Mann-Whitney U test). Correlations were calculated using Spearman rank correlation coefficient. All analyses were 2-sided and p values <0.05 were considered significant. Statistical analyses were performed using StatsDirect statistical software (version 2.7.8, Cheshire, United Kingdom).

## Results

Demographic details are shown in Table 1. BALF was available for all subjects and EBx for all control and 34 bronchiectasis subjects. Given significant differences between disease and control groups for age and use of ICS (either as monotherapy or as ICS/ LABA combination inhalers), multivariate regression analysis was undertaken (incorporating presence/ absence of bronchiectasis, ICS use, age, FEV_1_ as a percentage of the predicted value and gender) which showed no relationship between either of these variables and BALF IL-17A levels. The only significant independent predictor in this model was the presence of bronchiectasis (r = 0.3, p = 0.03).

**Table 1:** Subject demographics and disease characteristics.

		Control (n = 20)	Non-CF Bronchiectasis (n = 41)
Age (years)		36 (±11.8)	63 (±6.9)
Female—No. (%)		12 (60)	28 (68)
FEV_1_ (L)—pre-bronchodilator		3.52 (±0.83)	1.87 (±0.61)
FEV_1_ (L)—post-bronchodilator		3.61 (±0.83)	1.97 (±0.64)
FEV_1_% predicted (pre-bronchodilator)		97.9 (±12.6)	72.9 (±15.30)
FEV_1_% predicted (post-bronchodilator)		100.4 (±11.70)	76.8 (±14.42)
*Pseudomonas aeruginosa* in sputum—No. (%)		-	11 (27)
Other sputum pathogens—No. (%)			
	Normal flora only (no pathogens)	-	17 (41)
	*Haemophilus influenzae*	-	12 (29)
	*Stenotrophomonas maltophilia*	-	1 (2)
	Others	-	3 (7)
≥5 exacerbations in the prior year—No. (%)		-	16 (39)
24 hour sputum weight (g)—median (95% CI)		-	16.4 (13 to 20)
St George’s Respiratory Questionnaire*		-	39.4 (±14.4)
Leicester cough questionnaire* (LCS)		-	14.2 (±4)
6MWT (m)—median (95% CI)		-	510 (480 to 540)
Differential cell counts ** - median (IQR)			
	Total cell count (X 10^6^/ mL)	-	52.1 (31.2, 76.9)
	Macrophages (X 10^6^/ mL)	-	25.2 (1.1, 52.8)
	Macrophage %	-	42.6 (3.8, 75)
	Neutrophil (X 10^6^/ mL)	-	9.3 (3.4, 39.6)
	Neutrophil %	-	39.3 (6, 90.2)
	Lymphocytes (X 10^6^/ mL)	-	2.9 (0.5, 8.8)
	Lymphocytes %	-	6.8 (2.6, 14.9)
Medications—No.			
	Inhaled corticosteroids	0	5
	Combination inhalers (ICS/LABA)	0	17
	Inhaled LABA	0	2
	Inhaled SABA	0	17
	Inhaled anticholinergics	0	4
	Prednisolone	0	0
	Nebulised saline	0	2
	Bromhexine	0	2
Comorbidities -			
	Ischaemic heart disease	0	2
	Cerebrovascular disease	0	1
	Hypertension	0	9
	Diabetes mellitus	0	1

(Values are mean (±SD) unless otherwise indicated;

*lower scores on the LCS indicate worse cough symptoms, while lower scores on the SGRQ indicate better quality of life;

** Differential cell counts were performed on bronchiectasis BALF, and results exclude squamous and bronchial epithelial cells;

FEV_1_—forced expiratory volume in the first second; 6MWT—6 minute walk test; ICS—inhaled corticosteroids; LABA—long-acting β-agonist, SABA—short-acting β-agonist.)

### BALF

**Fig 1:**
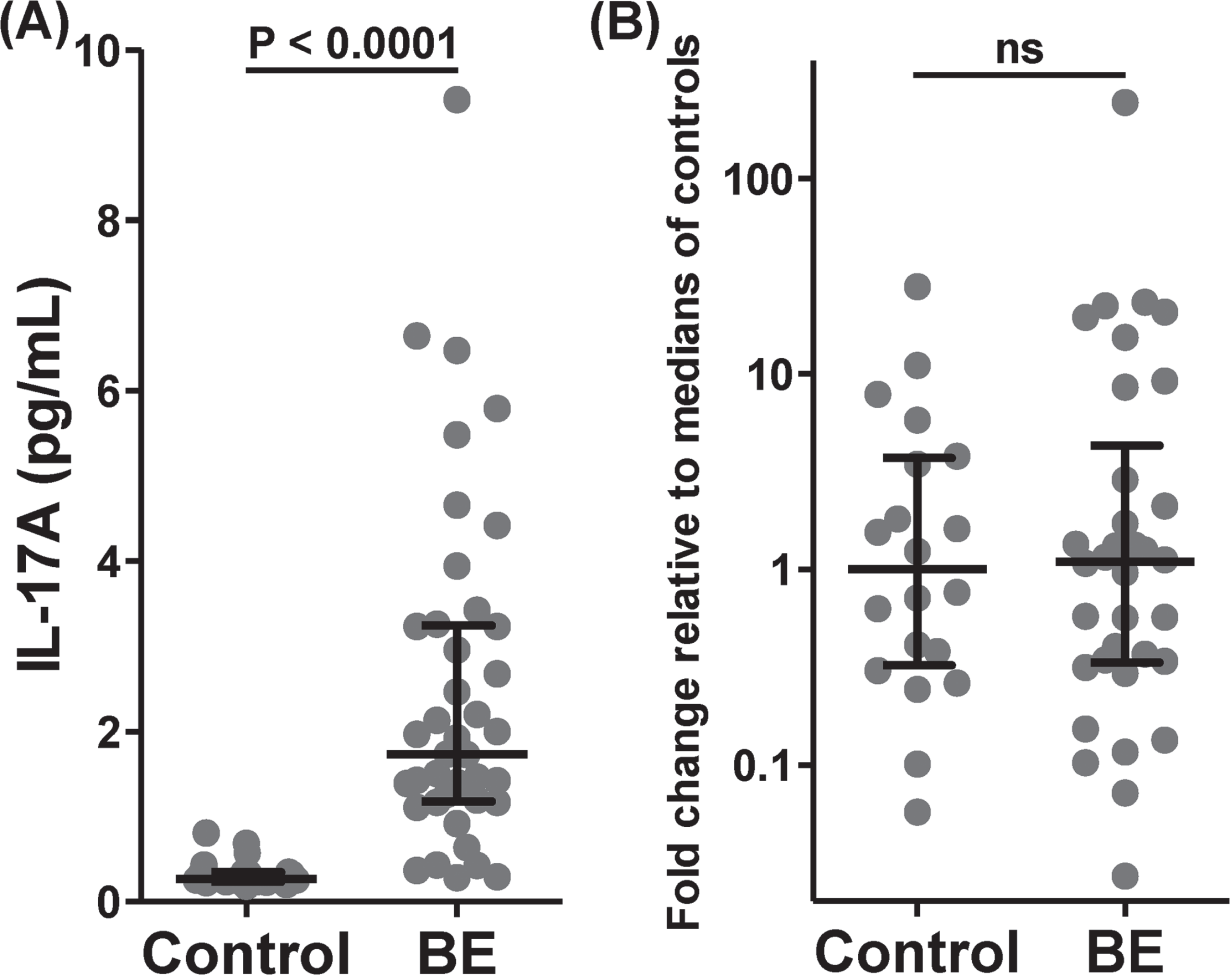
Airway luminal but not mucosal levels of IL-17A are significantly increased in adult non-CF bronchiectasis compared with healthy control subjects. (A) Bronchoalveolar lavage fluid levels of IL-17A comparing bronchiectasis (n = 41) and healthy control (n = 20) subjects; (B) Gene expression of IL-17A in endobronchial biopsies from bronchiectasis (n = 34) and healthy control (n = 20) subjects. (Cont—control, BE—bronchiectasis; Box and whisker plots display median and interquartile ranges; p values are by Mann-Whitney U test).

Median (IQR) levels of IL-17A were highly significantly increased in non-CF bronchiectasis (1.73 (1.19, 3.23) compared with control BALF (0.27 (0.24, 0.35) pg/mL, median difference 1.45, 95% CI 1.05 to 2.21, p<0.0001, Fig 1A). There were no significant differences in IL-17A levels within bronchiectasis subjects according to concurrent BALF culture results (*P*. *aeruginosa* (n = 9), 2.20 (1.39, 3.26), *Haemophilus influenzae* (n = 11) 1.46 (1.17, 2.46), normal respiratory flora only (n = 14) 1.85 (0.43, 4.42), p = 0.49 by K-W). Surprisingly, there were no differences in IL-17A levels between subjects with positive BALF bacterial culture (n = 27) (1.73 (1.26, 3.23)) and those with negative cultures (n = 14)(1.85 (0.43, 4.42), p = 0.68). Levels of IL-17A from bronchiectasis patients with chronic *P*. *aeruginosa* infection (2.58 (1.56, 3.34) n = 8) did not differ significantly from those with intermittent (1.90) (1.17, 3.94) n = 7) or no *P*. *aeruginosa* infection (2.14 (0.92, 2.68) n = 26, p = 0.26 by K-W). Finally, in contrast to the recent publication in CF children [12], bronchiectasis adults without chronic *P*. *aeruginosa* infection at baseline who subsequently cultured *P*. *aeruginosa* within 12 months did not have significantly higher levels of IL-17A (n = 10) (2.33 (1.11, 4.66) than those who did not (n = 23) (1.51 (1.17, 2.46), 95% CI for median difference -2.69 to 0.8, p = 0.64).

**Fig 2:**
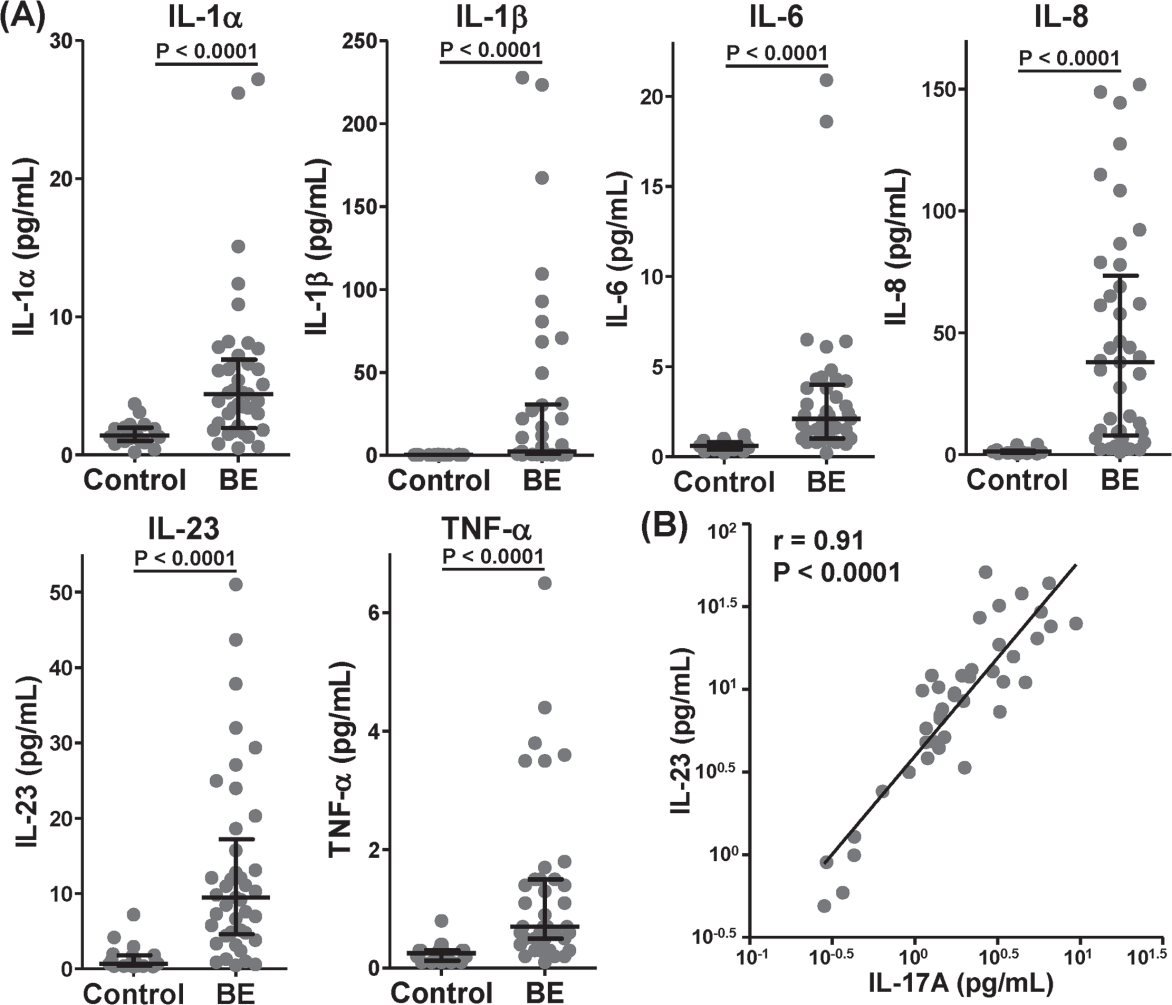
Bronchoalveolar lavage fluid levels of Th17 pathway cytokines and chemokines are significantly increased in adult non-CF bronchiectasis. (A) All Th17 pathway mediators are significantly increased in non-CF bronchiectasis (n = 41) compared with healthy control (n = 20) subjects. (B) Bronchoalveolar lavage fluid levels of IL-17A correlate strongly with all Th17 pathway mediators, but most strongly with IL-23 (n = 61). (Cont—control, BE—bronchiectasis; Box and whisker plots display median and interquartile ranges; p values are by Mann-Whitney U test).

Furthermore, IL-17A levels in BALF did not correlate with baseline percent-predicted FEV_1_, SGRQ, LCS and did not differ significantly between those with a 12 month history of ≥ 5 exacerbations versus those with <5. BALF IL-17A levels correlated significantly but weakly with BALF neutrophils (r = 0.35, p = 0.04), and strongly with all measured Th17 pathway cytokines (r >0.83 for all except IL-1α 0.68) with the strongest correlations to IL-23 (r 0.91, p<0.0001, Fig 2), TNF-α (r 0.90, p<0.0001) and IL-6 (r = 0.85, p<0.0001).

Compared to normal controls, subjects with non-CF bronchiectasis also had highly significantly elevated BALF levels of all measured Th17 pathway cytokines (IL-1α, IL1β, IL-6, IL-8, IL-23 and TNF-α (p<0.0001 for all, Fig 2).

**Fig 3:**
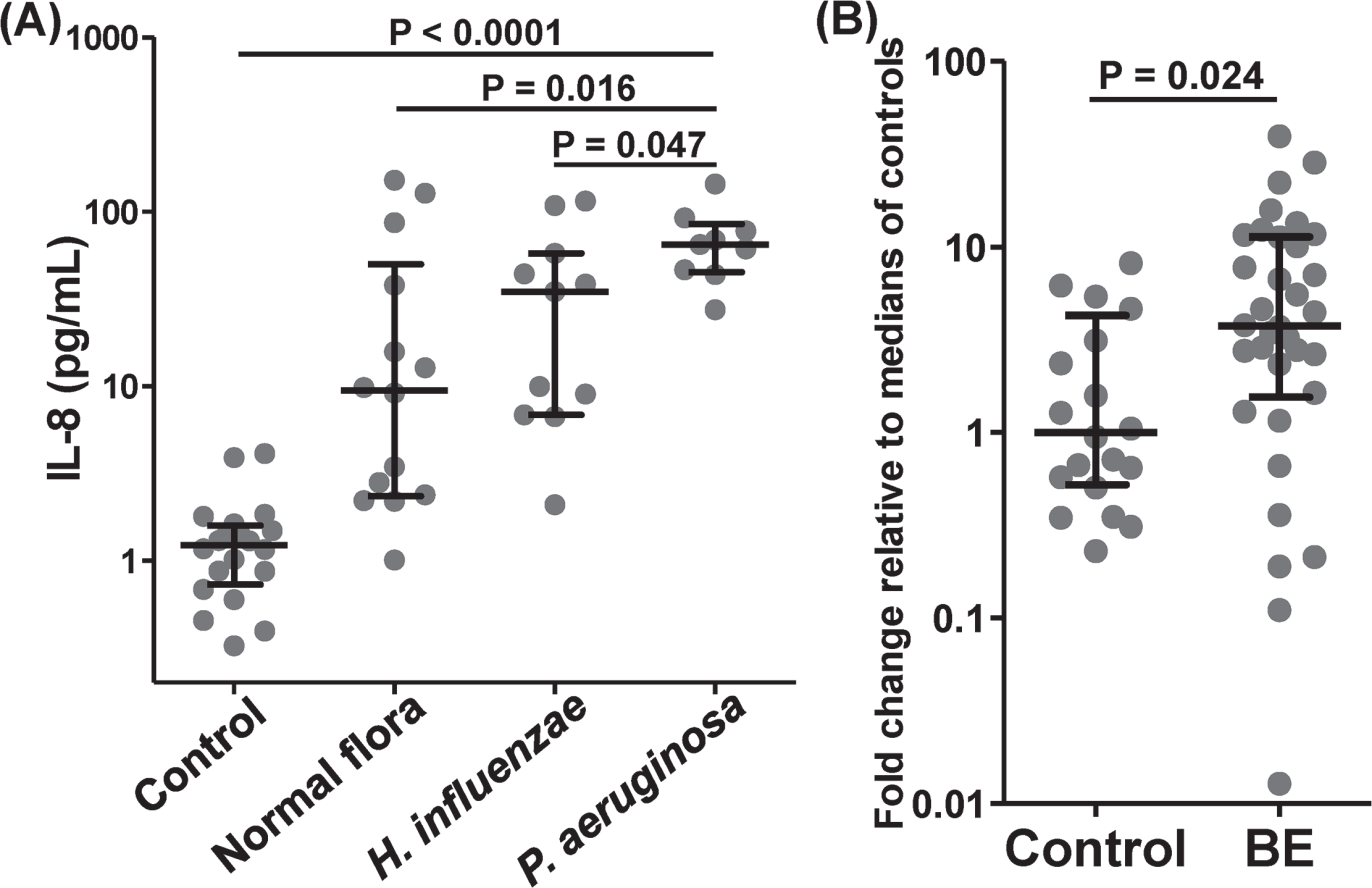
Airway luminal and bronchial mucosal levels of IL-8 are increased in non-CF bronchiectasis and relate to airway microbiology. (A) Bronchoalveolar lavage IL-8 levels are significantly increased in bronchiectasis subjects according to concurrent BALF infection by *P*. *aeruginosa* (n = 9) compared to *H*. *influenzae* (n = 11), normal respiratory flora only (n = 14) and healthy controls (n = 20). (B) Gene expression of IL-8 is significantly increased in endobronchial biopsy tissue from adult non-CF bronchiectasis (n = 34) than control (n = 20) subjects. (Cont—control, BE—bronchiectasis; Box and whisker plots display median and interquartile ranges; p values by Mann-Whitney U test).

Of these other cytokines and chemokines, only IL-8 and IL-1α showed differences according to BALF microbiology. IL-8 levels were significantly higher in those with positive (n = 27) (44.04 (14.66, 78.77) than negative BALF bacterial cultures (n = 14) (9.47 (2.38, 37.89), 95% CI 1.88–51.38, p = 0.03). Levels of IL-8 were also significantly elevated in those with *P*. *aeruginosa* in concurrent BALF (n = 9) (64.96 (46.4, 77.9) compared to those without *P*. *aeruginosa* (n = 32) (15.23 (5.77, 59.81), 95% CI 8.54 to 59.11, p = 0.019), those with normal respiratory flora cultured only (n = 14) (9.47 (2.38, 37.89), p = 0.016) and those with *H*. *influenzae* (n = 11) (34.85 (6.84, 57.75), 95% CI 2.4 to 59.9, p = 0.047, Fig 3). Interestingly, levels of IL-1α were numerically lower in those with BALF *P*. *aeruginosa* (n = 9) (20.25 (16.76, 28) than *H*. *influenzae* (n = 11) (32.18 (26.6, 70.18), however this trend was not statistically significant.

### Exploratory analyses for IL-8 and IL-1α

Given these IL-8 and IL-1α findings according to BAL microbiology, and although not part of our original intended evaluation, we undertook further evaluations of relationships between these 2 cytokines according to concurrent sputum microbiological results. Chronic pulmonary infection was defined as all sputum cultures being positive for the stated organism in the 6 months prior to enrolment, including each culture from sputum and BALF samples collected at screening and visit 1 for the BLESS study, and was hierarchical (*P*. *aeruginosa*>*H*. *influenzae* >others >normal flora only). Intermittent infection was defined where the organism was not cultured in every such specimen. ‘Baseline’ infection status was defined as per the BLESS study[14]—subjects with *P*. *aeruginosa* cultured at either the screening or visit 1 sputum sample were classified baseline *P*. *aeruginosa* positive, similarly for *H*. *influenzae* (if no *P*. *aeruginosa* cultured) and if no pathogens cultured in either then baseline ‘normal flora’. These investigations raise the possibility of a divergence of inflammatory stimulation according to specific microbiology. BALF IL-8 levels in subjects with chronic pulmonary infection by *P*. *aeruginosa* (n = 8) (63.08 (38.45, 73.4) did not differ from those with intermittent *P*. *aeruginosa* (n = 7) (46.43 (12.78, 92.22), but were significantly higher than levels in subjects without sputum *P*. *aeruginosa* (n = 26) (45.40 (3.44, 57.75), 95% CI 3.64 to 59.81, p = 0.043). IL-8 levels were significantly higher in subjects who cultured *P*. *aeruginosa* at any time during the 12 month study (n = 20)(53.82 (30.33,82.18) than those who did not (n = 21) (43.82 (3.44, 40.05), 95% CI 2.76 to 52.18, p = 0.03), however levels did not predict subsequent *P*. *aeruginosa* infection in subjects without chronic *P*. *aeruginosa* at baseline (subsequent *P*. *aeruginosa* (n = 10) 54.15 (6.84, 92.22) vs no subsequent *P*. *aeruginosa* (n = 23) 12.78 (3.44, 44.04), p = 0.21).

IL-1α levels were significantly higher in subjects with *H*. *influenzae* (n = 11) (35.86 (29.2, 79.3) than subjects with *P*. *aeruginosa* (n = 12) (19.87 (15.7, 31.55), 95% CI 2.98 to 46.9, p = 0.019) at baseline and in subjects with chronic *H*. *influenzae* infection (n = 8) (31.58 (29.21, 38.03) compared with chronic *P*. *aeruginosa* (n = 9) (19.87 (16.95, 27.88), 95% CI 1.21 to 42.05, p = 0.036).

BALF IL-8 levels were significantly correlated with BALF neutrophil counts (r = 0.67, p<0.0001), SGRQ symptoms scores (r = -0.48, p = 0.002) and LCS (r = 0.4, p = 0.01). BALF IL-1α levels were significantly correlated with SGRQ total scores (r = -0.33, p = 0.03), SGRQ symptoms (r = -0.38, p = 0.015) and LCS (r = 0.37, p = 0.017). Neither cytokine showed significant correlations with FEV_1_ or exacerbation frequency.

### Endobronchial biopsies

Gene expression of IL-17A in endobronchial biopsies did not differ between bronchiectasis and normal control subjects (median difference 0.03, 95% CI -0.66 to 0.77, p = 0.95, see Fig 1). However bronchiectasis subjects had significantly higher gene expression relative to the median of healthy controls for IL1β (4.12 (1.24, 8.05) vs 1 (0.13, 2.95), 95% CI 0.05 to 4.07, p = 0.04) and IL-8 (3.75 (1.64, 11.27) vs 1 (0.54, 3.89), 95% CI 0.32 to 4.87, p = 0.02, Fig 3).

## Discussion

The current study confirms, for the first time, significant airway luminal activation of the Th17 pathway in established adult non-CF bronchiectasis, in particular demonstrating significant elevations of BALF IL-17A and IL-23. However, in contrast to the recent publication in children with non-CF bronchiectasis, adults with established disease did not demonstrate significantly increased expression of IL-17A in endobronchial biopsies (assessed by gene expression rather than immunohistochemistry) [11]. Additionally, in contrast to data in CF children, BALF IL-17A levels did not predict subsequent *P*. *aeruginosa* infection [12]. Furthermore, we did not observe significant relationships between IL-17A levels and concurrent airway microbiology. Rather, our results showed increased IL-8 levels in association with *P*. *aeruginosa* infection and increased IL-1α levels in those with *H*. *influenzae* infection, suggesting both greater importance of these neutrophil-related mediators than IL-17A in established, chronic airway infection, and the possibility of divergent inflammatory activation according to specific microbiology. Our data support the proposal that innate, neutrophil-rich inflammation is a more important contributor to airway mucosal inflammatory pathophysiology in established adult non-CF bronchiectasis than a specific Th17 pathway response.

It is possible that our failure to demonstrate a more important role for the Th17 pathway represents relative underpowering; however, we were able to demonstrate the importance of IL-8 and IL-1 with this sample. Furthermore, these data are cross-sectional in nature and the disease and control groups were not matched, with significant differences for both age and inhaled corticosteroid use. However multivariate analyses did not identify any relationships between either of these variables and BALF IL-17A levels, with presence of bronchiectasis the only variable that predicted IL-17a levels. Additionally, we sampled a relatively large patient population for a study of this kind, and our bronchiectasis subjects, nested within our rigorously conducted, double-blind, randomised controlled BLESS study, were very well clinically characterized. Other strengths of the study include the evidence of internal validity provided by the strong relationships between all Th17 pathway cytokines.

Elevated levels of sputum IL-17A have previously been described in adult CF subjects [16,17], although only one of those studies had a comparator control group [16]. In that study, while sputum IL-17A gene expression was readily detected, the protein itself was unable to be measured in any of the controls and 3 (of 16) CF subjects. Additionally, IL-17 levels in bronchial mucosal tissue were not evaluated, and IL-23 was unable to be quantified at protein level [16]. Difficulty in measuring IL-23 protein levels in respiratory secretions has been reported recently [18,19]. Whether our ability to detect these cytokines in all healthy and bronchiectasis subjects reflects differences in sample processing methods (eg our use of protease inhibition) is not clear. Nonetheless, the demonstration of significantly elevated BALF IL-23 protein levels compared to controls is, to our knowledge, the first such data in human airways disease and confirms involvement of this key Th17 cytokine in airway inflammation in adult non-CF bronchiectasis. Additionally, our finding that the cytokine most strongly correlated with IL-17 levels was IL-23 is consistent with the ‘central role’ that IL-23 is believed to play in the Th17 pathway and Th17 cell maturation within the lung [20].

Our findings extend those of prior studies establishing the importance of neutrophilic inflammation in adult bronchiectasis [5,6,21,22]. The most comprehensive BALF study in adult non-CF bronchiectasis [5] demonstrated increased levels of neutrophil elastase, myeloperoxidase, TNF-α, IL-6 and IL-8 but not IL1β compared to controls. In addition to revealing upregulation of IL-1α, IL-17A and IL-23 and confirming increased BALF levels of IL-6, IL-8 and TNF-α, our data also demonstrate highly significant increases in BALF IL-1β in contrast to that earlier study. This difference may reflect the milder disease of subjects in that study, with an average FEV_1_ 79% predicted and a higher proportion of subjects negative for potential pathogens (52% vs 34% in our study).

The current data demonstrate highly significant increases in all Th17 pathway-associated chemokines and cytokines measured in the airways of adult bronchiectasis subjects, including pathway effectors (IL-17A, IL-6, IL-8 and TNF-α) and regulators (IL-1α, IL-1β, IL-6 and IL-23). However, while gene expression of both IL-1β and IL-8 was significantly upregulated in the bronchial mucosa of non-CF bronchiectasis subjects, IL-17A was not, possibly reflecting the scarce relative abundance of the cells of origin in the mucosa. Interestingly, in spite of demonstrating significant increases in the abundance of Th17 T cells in biopsies, Tan and colleagues did not demonstrate significant increases in BALF IL-17 in non-CF bronchiectasis compared to control children [11]. Differences in findings between the 2 studies are likely to reflect differences in clinical status (our subjects were all clinically stable, versus all paediatric bronchiectasis subjects being sampled at the time of exacerbation), age (young children vs old adults) and degree of chronicity of airway infection. In addition to production by Th17 T cells, γδ cells, innate lymphoid cells and NK cells, IL-17 can be secreted by neutrophils [9, 11, 23]. Luminal neutrophils might be expected to be a relatively much greater source of IL-17A in old subjects with stable but established bronchiectasis, while mucosal IL-17A appears to predominate in childhood bronchiectasis subjects with earlier infection [11]. Consistent with this, we observed substantially higher neutrophil counts in BALF from our subjects (mean 21 X 10^6^/mL) than reported in the Tan paper (from Figure 5(C), approx. 150/mL)[11]. Furthermore, BALF IL-17A levels correlated significantly with BALF neutrophils but not with gene expression of IL-17A in endobronchial biopsies. Compartmentalization of IL-17A production (luminal vs. epithelial) in these subjects with established bronchiectasis also explains the apparent discrepancy between elevated protein levels of IL-17A in BALF but not gene expression in biopsies.

Prominent neutrophilia is part of the intense cellular infiltration of bronchial mucosa that has been shown in adult bronchiectasis [21], and neutrophilic inflammation is characteristic of acute Th17 pathway activation in the lungs [24]. Interestingly however, an IL-17-overexpressing murine model assessed from 5 months showed significant lung inflammation characterised by increases in lung macrophage and lymphocyte, but not neutrophil, infiltration [25]. IL-17A release in response to acute infection is predominantly from innate immune cells rather than Th17 cells [23] and neutrophils were shown to be the main cellular source of IL-17 in a murine model of inhalational anthrax [26]. Hence, we speculate that neutrophils are the primary source of airway IL-17A in the chronic airway inflammation characterizing adult non-CF bronchiectasis.

Our results suggest relatively greater importance of the neutrophil-related mediators IL-8 and IL-1α, than IL-17A, in adult non-CF bronchiectasis airway mucosal pathophysiology. Both IL-8 and IL1α, but not IL-17A, correlated with cough symptoms and quality of life measures. Furthermore, while there were no significant differences in BALF IL-17A levels between subjects according to important clinical markers, IL-8 levels were significantly increased in those with the presence of any potentially pathogenic microorganism and also *P*. *aeruginosa* infection specifically. Interestingly, those with *H*. *influenzae* had levels of IL-1α that were significantly higher than those with *P*. *aeruginosa*. The demonstration in sputum of significantly higher levels of neutrophilic inflammatory mediators in subjects with *P*. *aeruginosa* infection has recently been described in a large study [6]. However, we are unaware of any data suggesting either decreased IL-1α levels in subjects with *P*. *aeruginosa* (compared to *H*. *influenzae*) infection or the possibility of a divergence in the specific inflammatory response according to specific microbiology. This divergent inflammatory activation might explain the significantly higher rates of pulmonary exacerbation seen in subjects with *P*. *aeruginosa* than *H*. *influenzae* in the BLESS study [13,27,28]. For example, perhaps *P*. *aeruginosa* minimizes epithelial IL-1α (an intracellular cytokine mainly released following cell necrosis) activation and release in order to evade mucosal immune systems, analogous to its ability to inhibit macrophage IL-1β production via components of its Type 3 secretory system [29]. It must be recognised that some of these analyses (based upon concomitant sputum bacterial infection status) were post-hoc, performed when our initial analysis suggested the possibility of such a relationship according to BALF infection status. Further investigation of the specific inflammatory responses of bronchiectasis model systems to different microbial communities will be informative in this regard.

There is significant airway luminal activation of the Th-17 pathway in established, adult non-CF bronchiectasis. However, our results suggest relatively greater importance of non-antigen-specific innate neutrophil-dominated inflammation in the airway pathophysiology of this condition. We speculate that specific activation of the Th17 pathway is less important in the longstanding, chronic infection and inflammation that characterises adult bronchiectasis once neutrophilic inflammation has become established.

## Supporting Information

S1 Supporting Information Data Supplement. (DOCX)
