# Supplementary material for: Adult Non-Cystic Fibrosis Bronchiectasis Is Characterised by Airway Luminal Th17 Pathway Activation
Source: PLoS One. 2015 Mar 30;10(3):e0119325. doi: 10.1371/journal.pone.0119325 (PMC4379018; doi:10.1371/journal.pone.0119325)
Supplement: S1 Supporting Information — (DOCX) [file pone.0119325.s001.docx]

**Data Supplement**

Serisier DJ, Chen AC-H, Lourie R, Martin M, Rogers GB, Burr L, Hasnain SZ, Bowler SD, McGuckin MA

**Study design**

This was a cross-sectional evaluation of Th17 pathway activation in a group of stable subjects with non-CF bronchiectasis, nested within the randomised, placebo-controlled BLESS trial, compared with control subjects.[1] In addition, 12 month follow-up microbiology data were obtained for the non-CF bronchiectasis subjects as they proceeded in the BLESS study. The study comprised 2 linked studies performed in parallel, as part of a prospective study designed to evaluate the airway pathophysiology of non-CF bronchiectasis: the BLESS study in a subgroup of subjects with non-CF bronchiectasis; and the linked control group study. This study was performed between October 2008 and December 2011, at the Mater Adult Hospital, an Australian university teaching hospital, through the respiratory medicine department which is also a regional adult cystic fibrosis centre. Non-CF bronchiectasis subjects were a subgroup of participants from the BLESS study who met additional selection criteria as detailed below. The control subjects were screened from healthy volunteers identified at the hospital and the Mater Research Institute, who otherwise met selection criteria as detailed below. Control subjects were identified in response to advertisements placed in the hospital and at the Mater research Institute. The control group was ultimately comprised of hospital staff members, students placed at the hospital, and laboratory research staff. Control subjects underwent investigations as detailed, but did not undergo any subsequent follow-up.

Non-CF bronchiectasis subjects recruited to the current study represented a subgroup of subjects from the BLESS study. Additional selection criteria for this subgroup are provided starting on page 6, but in essence they represented subjects in whom bronchoscopy could be performed with acceptable safety and who did not have any evidence of non-bronchiectasis related airway disease (ie no evidence of asthma, atopy, smoking/ COPD, immune deficiency, etc). The selection criteria for the BLESS study itself are first provided below.

**Selection criteria for the BLESS study [1]:**

***Non-CF bronchiectasis subject inclusion criteria:***

1. Able to provide written informed consent.

2. Confirmed diagnosis of bronchiectasis by HRCT within 3 years.

3. Airways obstruction on spirometry (ratio FEV_1_/ FVC <0.7) and FEV_1_ ≥25% predicted.

4. Chronic productive cough with at least 5 mLs sputum production per day.

5. At least two exacerbations of bronchiectasis requiring either oral or intravenous supplemental antibiotic therapy (of at least 7 days on each occasion) in the prior 12 months.

6. Aged 20-85 inclusive.

7. Clinically stable for at least four weeks (defined as no symptoms of exacerbation, no requirement for supplemental antibiotic therapy, and FEV1 within 10% of best recently recorded value where available).

***Exclusion criteria***

1. Bronchiectasis as a result of CF or focal endobronchial obstruction.

2. Currently active tuberculosis or non-tuberculous mycobacterial (NTM) infection. Subjects with evidence of prior pulmonary NTM infection could be included only if they have completed a course of therapy that is deemed successful on the basis of negative NTM cultures following cessation of therapy. All subjects required a negative NTM culture prior to screening.

3. Any symptoms or signs to suggest recent deterioration in respiratory disease, including exacerbation of pulmonary disease (as previously defined) in the preceding 4 weeks.

4. Any change to medications in the preceding 4 weeks.

5. Prescription of either oral or intravenous antibiotic therapy in the preceding 4 weeks.

6. Cigarette smoking within the preceding 6 months.

7. Any history of malignant arrhythmia (unless in the immediate post-myocardial infarction period and not requiring any regular therapy) or QTc prolongation on baseline ECG.

8. Any of the following within the three (3) months prior to enrolment:

- Acute MI
- Acute CVA
- Major surgery

9. History of any of the following:

- Active malignancy (excepting non-melanoma skin malignancies that have been treated and considered cured)
- Listed for transplantation
- Any other significant active illness likely to affect the patient’s survival within 12 months
- Receiving long-term domiciliary oxygen therapy

10. Allergy to macrolide antibiotics, other than minor, dose-related gastrointestinal intolerance that would not be anticipated to recur with low-dose erythromycin.

11. Any prescription or receipt of long-term macrolide antibiotics, or receipt of a treatment course within 4 weeks.

12. Predominant diagnosis of emphysema (rather than bronchiectasis) on HRCT scan of the chest.

13. Requirement for supplemental oxygen therapy.

14. Inability to complete required study procedures for whatever reason (including 6 minute walk test, hypertonic saline sputum induction).

15. Respiratory symptoms (including cough, sputum production, recurrent exacerbations) not predominantly the result of bronchiectasis in the opinion of the PI; where treatable causes for exacerbations existed, these were treated before considering trial enrolment.

***Excluded medications***

1. Macrolide antibiotics – long-term macrolide use was an absolute exclusion, however subjects who had received a short duration (less than 6 weeks) treatment course were eligible provided they had at least 4 weeks washout.
2. Long term oral antibiotic administration for infection prophylaxis (eg doxycycline).
3. Any other intravenous or oral antibiotic within 4 weeks.
4. While erythromycin in the current study was administered in a low dose, possible drug interactions in all patients entering the study were considered. Subjects using the following medications were not eligible for the study:
   - ergotamine or dihydroergotamine
   - triazolam/ alprazolam
   - sildenafil
   - azole antifungals (ketoconazole, itraconazole, fluconazole)
   - disopyramide
   - quinidine

Concomitant HMG-CoA reductase inhibitor (‘statin’) use was permitted, however subjects on high-dose statins (equivalent to 80 mgs daily of simvastatin) required dose reduction by half at study entry.

Subjects prescribed diltiazem or verapamil were screened to ensure no evidence of clinically relevant increases in levels of these medications.

The following medications were permitted, provided they had been a regular medication for at least 6 months (with the requisite number of exacerbations whilst on this therapy):

1. Inhaled antibiotics in chronic, daily, stable dose.
2. Inhaled mucolytic therapies (hypertonic saline, mannitol, dornase alpha, N-acetylcysteine).
3. Oral mucolytics or expectorants.

**Additional selection criteria for the current study (bronchoscopy subgroup from BLESS):**

1. Never smokers.
2. No hypogammaglobulinaemia (levels performed at screening) or ciliary dysfunction.
3. Not asthmatic and without evidence of atopy on bloods (RAST/ s.IgE).
4. FEV_1_>40% predicted.
5. Not warfarinised or have other issues in relation to anticoagulation or bleeding diatheses that make them unsuitable for endobronchial biopsy due to bleeding risks.
6. No clinically significant ischaemic or myocardial disease that increases the risk in relation to bronchoscopy.
7. No hypercapnoea or evidence of overlap syndrome.

**Selection criteria for the normal control subjects:**

***Inclusion criteria***

1. Age 18 – 85 years.

2. Normal subjects without any identifiable airway disease.

3. Capable of providing written, informed consent to participate.

4. Lifelong non-smokers (< or = 2 pack year history of smoking).

5. Normal spirometry (defined as values for FEV_1_, FVC and ratio of FEV_1_:FVC all lying within the normal predicted range according to age and height, no significant acute response to inhaled bronchodilator and no evidence of small airways obstruction on flow-volume loops).

***Exclusion criteria:***

1. Smoking history >2 pack year history of tobacco smoking.
2. Any smoking history of other substances.
3. History of any chronic respiratory disease including asthma, chronic obstructive pulmonary disease/ COPD, bronchiectasis, etc, or pneumonia within 8 weeks of bronchoscopy.
4. Any respiratory tract infection (including upper respiratory tract infection) within 4 weeks of bronchoscopy.
5. Medications: prescription of any antibiotic, antihistamine, corticosteroid, mast cell stabilising therapy, theophylline or any inhaled therapy within 4 weeks of bronchoscopy.
6. Any condition with the potential to increase the risks of bronchoscopy/ bronchoalveolar lavage or endobronchial biopsy, including (but not limited to) bleeding diathesis (platelet count < 150, abnormal coagulation profile, warfarin therapy), any unstable medical condition (eg acute cardiac or cerebral ischaemic event within 3 months, active malignancy), any significant illness likely to impact upon survival of the patient within 12 months, requirement for domiciliary oxygen.
7. Positive bronchial challenge test or chronic abnormalities detected on CT scan of the chest.

**Procedures**

*Bronchoscopy procedure*

Subjects were fully informed about the potential risks of the procedure and provided written consent. Bronchoscopy was performed as an outpatient procedure in the endoscopy unit of the operating theatres of the Mater Adult Hospital, using an Olympus flexible fibre-optic bronchoscope according to the safety standards of the Thoracic Society of Australia and New Zealand,[3] with details of the research bronchoscopy procedure adapted from prior methods.[4-6] Subjects fasted for 6 hours before the procedure. The procedure was performed transorally, under light sedation using intravenous midazolam and fentanyl to ensure patient comfort. Topical lignocaine was applied to the vocal cords and bronchi by instillation through the bronchoscope. Where possible, no suction was employed prior to performing BAL in the target lobe. After wedging in a right middle lobe bronchus, BAL was performed. In non-CF bronchiectasis subjects without significant right middle lobe bronchiectasis (on HRCT), an alternative lobe with bronchiectatic change was selected according to a hierarchy – lingula, upper lobe (right or left), apical segment lower lobe.

One hundred and forty (140) mLs of warmed sterile saline was gently instilled, followed by gentle aspiration through the bronchoscope’s suction channel, and aspirated BALF was pooled.

Following bronchoalveolar lavage, 8-10 endbronchial biopsies were then taken from subsegmental carinae of the lower lobes using Boston scientific Radial Jaw 3 single-use biopsy forceps (diameter 1.8 mm), starting at 5^th^ order airways and working proximally as far as the 3^rd^ order bronchi if necessary (bifurcation of segmental and subsegmental bronchi). Subjects were observed for 2 hours after the bronchoscopy before being allowed home.

*Microbiology*

Sputum and BALF processing for culture and sensitivity testing were performed in the Division of Microbiology, Mater Pathology. Sputum or BALF was transported to the laboratory within 60 min of collection and processed within 3 h (refrigerated at 4°C in the interim). The most purulent portion of the sputum specimen was selected and streaked directly onto horse blood agar (HBA), MacConkey agar, chocolate agar supplemented with bacitracin (CHOC-B), Sabouraud agar, and mannitol salt agar. Plates were incubated at 37°C (HBA and CHOC-B in CO_2_ and CHOC-B anaerobically and the rest in O_2_) for at least 48 h and examined daily. Organism identification incorporated a combination of typical morphology, species specific manual tests (eg. catalase testing, coagulase testing, Gram stain, pigment production etc.), API, and Vitek identification.

*Differential cell counts in BALF*

BALF was incubated in a shaking water bath at 37° C for 5-10 min, and gently mixed using a transfer pipette at 5-min intervals. A further three times the volume of phosphate-buffered saline (Dulbecco's; Gibco BRL, Grand Island, NY) was added and the mixture incubated again in the 37° C shaking water bath for another 5-10 min. Ten microliters of the homogenized sputum samples, mixed with Trypan Blue, was used to calculate total cell counts, using a standard hemacytometer. A further 0.25-0.50 ml of both samples was used to prepare cytospin slides for differential cell counts, centrifuging at 750 rpm for 5 minutes. After staining the slides with Wright’s stain, 300 cells were counted and cell differentials calculated.

*BALF processing for soluble mediator assessments*

BALF was centrifuged at 500 g for 5 min at 4°C to pellet large cellular debris, then supernatant centrifuged at 16,000 g for 10 min at 4°C, and the resultant supernatant collected and filtered through a 0.2 μm filter syringe before storage at -80°C, in aliquots with or without protease inhibitors (Complete Protease Inhibitor Cocktail Tablets, Roche, Basel, Switzerland).

*Gene expression data from endobronchial biopsies*

Two endobronchial biopsies collected for subsequent RNA extraction were placed into a tube containing 0.5 mL of RNA Later solution (Life Technologies, Carlsbad, CA) and transferred on ice for storage at -80 C. RNA was extracted from biopsies using an on-column method as per manufacturer’s instructions for the *RNeasy* RNA extraction kit (QIAGEN, Hilden, Germany). RNA quality was assessed by detecting the 18S and 28S rRNA using electrophoresis method as per the manufacturer’s instruction for the Experion RNA StdSens Analysis Kit (Bio-Rad, Hercules, CA). Due to low RNA yields from airway biopsies, whole transcriptome amplification was performed as per the manufacturer’s instruction for the WT-Ovation RNA Amplification Kit (NuGen, San Carlos, CA), and the end product in this protocol was cDNA.

cDNA synthesis for the non-amplified RNA samples was performed as per the manufacturer’s instruction for the iScript cDNA synthesis kit (Bio-Rad, Hercules, CA). 1 ug RNA from each sample was used as template in each cDNA synthesis reaction.

RNA was extracted from biopsies or HBEC, and gene expression of IL-1β, IL-8, IL-13 and IL-17a measured using the TaqMan detection system as per the manufacturer’s instructions (Microfluidic Cards, Life Technologies, Carlsbad, CA). The average CT of three house-keeping genes (β2-microglobulin, β-actin and Cyclophilin A) was used for standardization.

**Data analysis/ outcome measures**

The primary analyses of interest in the current study related to Th17 pathway cytokine levels in BALF and gene expression in EBx, comparing bronchiectasis and control groups.

Our additional predefined analyses included:

1. Evaluation, in the bronchiectasis subjects, of differences in all measured BALF Th-17 pathway cytokines according to concurrent BALF infection status (BALF culture positive vs. culture negative; *P. aeruginosa* vs. *H. influenzae* vs. ‘normal respiratory flora’ infection), to explore relationships between infection and BALF cytokines.
2. The ability of baseline BALF IL-17A levels to predict the subsequent development of *P. aeruginosa* infection in those without chronic infection at baseline (the analysis was a comparison of baseline BALF IL-17A levels in those who developed such infection in the subsequent 12 months to those who did not).
3. Correlations between BALF IL-17A levels and the following demographic/ clinical measures: post-bronchodilator percent-predicted FEV_1_, number of pulmonary exacerbations reported in the preceding 12 months (<5 vs ≥5), QOL scores (SGRQ total and SGRQ symptoms), cough symptom scores (LCS), 24 hour sputum weight.
4. Correlations between BALF IL-17A levels and BALF neutrophil counts and all measured Th17 pathway cytokines.

Exploratory analyses:

The lack of any significant relationships between microbiology and IL-17A, but interesting relationships instead for IL-8 and IL-1 resulted in us undertaking further post-hoc, exploratory relationships of these cytokines. These exploratory evaluations included:

1. Comparisons of BALF levels of these 2 cytokines between subjects according to concurrently performed sputum microbiology results (chronic and ‘baseline’ infection by *P. aeruginosa*, *H. influenzae*, normal flora).
2. The ability of baseline BALF IL-8 levels to predict the subsequent development of *P. aeruginosa* infection (given that elevated IL-8 levels appear to be associated with this infection) in those without chronic infection at baseline (the analysis was a comparison of baseline BALF IL-8 levels in those who developed such infection in the subsequent 12 months to those who did not).
3. Correlations of BALF IL-8 and IL-1 levels to BALF neutrophil counts, post-bronchodilator percent-predicted FEV_1_, number of pulmonary exacerbations reported in the preceding 12 months (<5 vs ≥5), QOL scores (SGRQ total and SGRQ symptoms), cough symptom scores (LCS), 24 hour sputum weight.

**References**

1. David J Serisier, Megan L Martin, Michael A McGuckin, et al. Effect of long-term, low-dose erythromycin on pulmonary exacerbations among patients with non-cystic fibrosis bronchiectasis. The BLESS randomized, controlled trial. JAMA 2013
2. Paggiaro PL, Chanez P, Holz O, *et al*. Sputum induction. *Eur Respir J* 2002;**20**:Suppl 37, 3s-8s.
3. Wood-Baker R, Burdon J, McGregor A, *et al*. Fibre-optic bronchoscopy in adults – a position paper of the Thoracic Society of Australia and New Zealand. *Intern Med J* 2001;**31**:479-87.
4. Monton C, Torres A, El-Ebiary M, *et al*. Cytokine expression in severe pneumonia: a bronchoalveolar lavage study. *Crit Care Med* 1999;**27**:1745-53.
5. Hilliard JB, Konstan MW, Davis PB. Inflammatory mediators in CF patients. In Methods in Molecular Medicine 2002, vol 70: Cystic Fibrosis Methods and protocols. WR Skach (ed), Humana Press Inc, Totowa, NJ, USA
6. Hattotuwa K, Gamble EA, O’Shaughnessy T, *et al*. Safety of bronchoscopy, biopsy and BAL in research patients with COPD. *Eur Respir J* 2002;**122**:1909-12
